# Poor glycated haemoglobin control and adverse pregnancy outcomes in type 1 and type 2 diabetes mellitus: Systematic review of observational studies

**DOI:** 10.1186/1471-2393-6-30

**Published:** 2006-10-30

**Authors:** Melanie E Inkster, Tom P Fahey, Peter T Donnan, Graham P Leese, Gary J Mires, Deirdre J Murphy

**Affiliations:** 1 Division of Community Health Sciences, University of Dundee, MacKenzie Building, Kirsty Semple Way, Dundee, UK, DD2 4BF; 2 Department of General Practice, Royal College of Surgeons in Ireland, Mercer's Medical Centre, Stephen Street Lower, Dublin 2, Ireland; 3 Diabetes Centre, Ninewells Hospital and Medical School, Dundee, UK, DD1 9SY; 4 Maternal and Child Health Sciences, Ninewells Hospital and Medical School, Dundee, UK, DD1 9SY; 5 Department of Obstetrics and Gynaecology, Trinity College, University of Dublin, Ireland

## Abstract

**Background:**

Glycaemic control in women with diabetes is critical to satisfactory pregnancy outcome. A systematic review of two randomised trials concluded that there was no clear evidence of benefit from very tight versus tight glycaemic control for pregnant women with diabetes.

**Methods:**

A systematic review of observational studies addressing miscarriage, congenital malformations and perinatal mortality among pregnant women with type 1 and type 2 diabetes was carried out. Literature searches were performed in MEDLINE, EMBASE, CINAHL and Cochrane Library. Observational studies with data on glycated haemoglobin (HbA_1c_) levels categorised into poor and optimal control (as defined by the study investigators) were selected. Relative risks and odds ratios were calculated for HbA_1c _and pregnancy outcomes. Adjusted relative risk estimates per 1-percent decrease in HbA_1c _were calculated for studies which contained information on mean and standard deviations of HbA_1c_.

**Results:**

The review identified thirteen studies which compared poor versus optimal glycaemic control in relation to maternal, fetal and neonatal outcomes. Twelve of these studies reported the outcome of congenital malformations and showed an increased risk with poor glycaemic control, pooled odds ratio 3.44 (95%CI, 2.30 to 5.15). For four of the twelve studies, it was also possible to calculate a relative risk reduction of congenital malformation for each 1-percent decrease in HbA_1c_, these varied from 0.39 to 0.59. The risk of miscarriage was reported in four studies and was associated with poor glycaemic control, pooled odds ratio 3.23 (95%CI, 1.64 to 6.36). Increased perinatal mortality was also associated with poor glycaemic control, pooled odds ratio 3.03 (95%CI, 1.87 to 4.92) from four studies.

**Conclusion:**

This analysis quantifies the increase in adverse pregnancy outcomes in women with diabetes who have poor glycaemic control. Relating percentage risk reduction in HbA_1c _to relative risk of adverse pregnancy events may be useful in motivating women to achieve optimal control prior to conception.

## Background

Diabetes is the most common pre-existing medical condition complicating pregnancy in the United Kingdom (approximately four occurrences per 1000 pregnancies) [1]. It is known to have a substantial impact on maternal, fetal and neonatal outcomes. The presence of diabetes is said to increase the risk of congenital malformation (by ten-fold), the risk of stillbirth (by five-fold), and the risk of neonatal death (by three-fold) [2-7] These disappointing data are in contrast to the optimism of the 1989 St Vincent's Declaration that proposed as a five year target that the outcome of pregnancy should approximate that of the non-diabetic pregnancy [8].

A pivotal part of management is good diabetic control which is believed to reduce the incidence of pregnancy complications. Glycated haemoglobin (HbA_1c_) reflects long-term glycaemic control and is a more accurate and stable measure than fasting blood glucose levels [9]. It tracks well over time in individuals with diabetes and has less variability than fasting blood glucose.

Longer term glycaemic control in women with diabetes is critical to satisfactory pregnancy outcome. As organogenesis takes place in the first trimester of pregnancy, inadequate pre-conceptual glycaemic control is associated with an increased risk of congenital abnormality and spontaneous abortion [10,11].

Clinical management decisions are limited by a dearth of randomised trial data due to ethical reasons and current practice must rely on the findings of high quality observational studies.

The objective of the study was to perform a systematic review of observational studies to investigate and quantify the risk of adverse pregnancy outcomes in pregnant women with diabetes in relation to glycaemic control, whether poor or optimal.

## Methods

### Study design

We systematically reviewed observational studies of glycated haemoglobin and pregnancy outcomes in women with diabetes mellitus.

### Study selection

**Figure 1:**
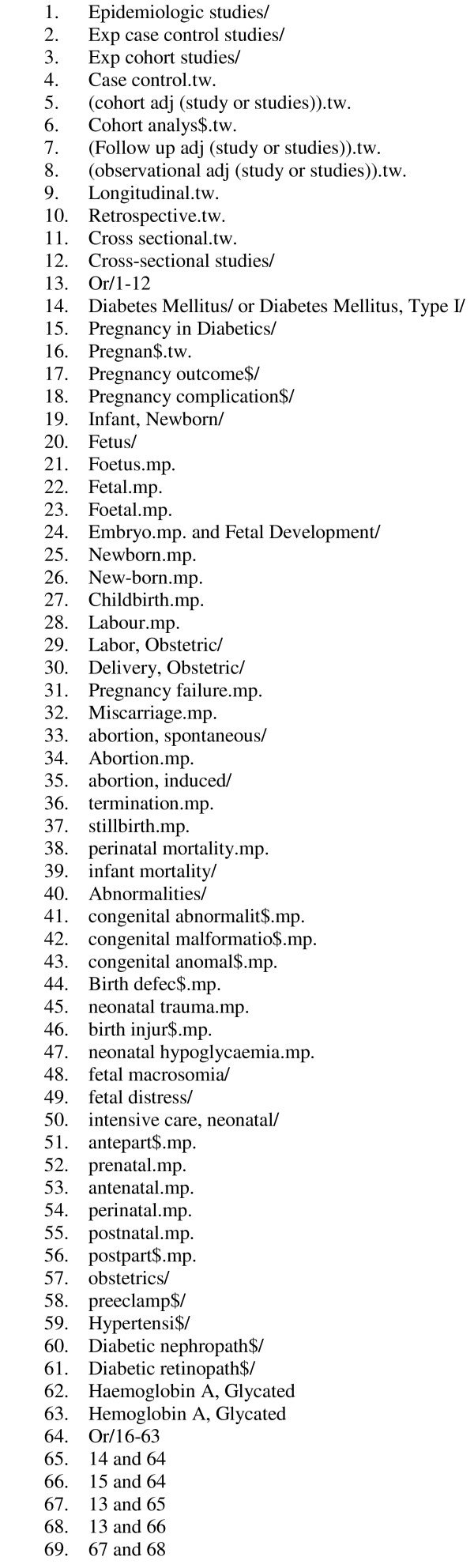
Search strategy.

We searched the MEDLINE database for articles published in English from 1966 to January 2005 by using Medical Subject Heading terms and text words related to pregnancy, diabetes mellitus, glycaemic control and glycated haemoglobin (Figure 1 contains the full text of the search strategy). We also searched EMBASE, CINAHL and the Cochrane Library.

We reviewed all abstracts obtained from our search for relevance. We manually reviewed bibliographies and review articles for additional citations and obtained the full text of potentially relevant articles.

Our pre-specified inclusion criteria were as follows: 1) published observational studies; 2) studies that examined pregnancy outcomes in women with type 1 and type 2 diabetes; and 3) studies that reported a measure of glycated haemoglobin and had clearly categorised pregnancy outcomes according to poor and optimal glycaemic control using a cut-off point. Case-control studies were included in the review only if there was clear distinction between optimal and poor glycaemic control in relation to outcomes in the cases (patients with diabetes). Control data from case-control studies were not used in this review. We excluded studies if they included women with gestational diabetes.

### Data abstraction

One investigator reviewed each article that met the selection criteria and abstracted the data by using standardised data abstraction forms. Information was collected on study design, country of study, time-period of study, glycaemic control groups, method of measuring glycated haemoglobin, and timing of glycaemic control measurement. Data abstracted were age, parity, smoking, duration of diabetes, pre-pregnancy planning, folic acid consumption, presence of microvascular complications, pre-pregnancy insulin dose, sample size, type of outcome or outcomes, main results, statistical methods and variables, if any, which were included in the adjusted model or models.

For each study that met our inclusion criteria, we abstracted relative risks and odds ratios for the association between adverse pregnancy outcomes and poor vs. optimal glycaemic control if they were stated. If not, then the relative risks and odds ratios were calculated from information stated in each study.

A range of outcomes were investigated, however due to the quality of data we focussed on congenital malformations, miscarriage and perinatal mortality (including stillbirths and neonatal deaths).

A quality of reporting of meta-analyses of randomised controlled trials (QUOROM) checklist was carried out [see Additional file 1]. Quality assessment was modified to suit a meta-analysis of observational studies rather than randomized controlled trials, examining patient selection, data extraction methods, losses to follow up, and confounding.

### Statistical analysis

In the primary analysis, glycated haemoglobin (HbA_1c_) was the principal 'exposure' of interest. HbA_1c _was categorised into poor and optimal control. Dichotomous outcomes are expressed as odds ratios and 95% confidence intervals are calculated. A test of heterogeneity, Cochran's Q-test, was performed for each outcome and if no heterogeneity was present, a fixed-effects meta-analysis was performed. If heterogeneity was marked, random effects models were performed.

For studies that reported the mean and SD of HbA_1c _we estimated the effect of a 1-unit percent change in HbA_1c_, assuming a normal distribution for HbA_1c _values. We calculated the 25^th ^and 75^th ^percentiles and divided the log relative risk by the difference of these 2 values to give an estimate of the effect of a 1-percent change in HbA_1c _[12]. We did not pool data from individual studies for these analyses as the measurement of HbA_1c _differed between centres.

We assessed publication bias where possible by using the Egger test [13] and funnel plots which graphically display the magnitude of the effect estimate by the inverse variance of the study. Sensitivity analyses assessed the relative influence of each study by omitting one study at a time to assess the influence of the single study on the pooled estimate.

Statistical analyses were conducted using StatsDirect [14] and Stata Version 8 software.

## Results

### Search results

**Figure 2:**
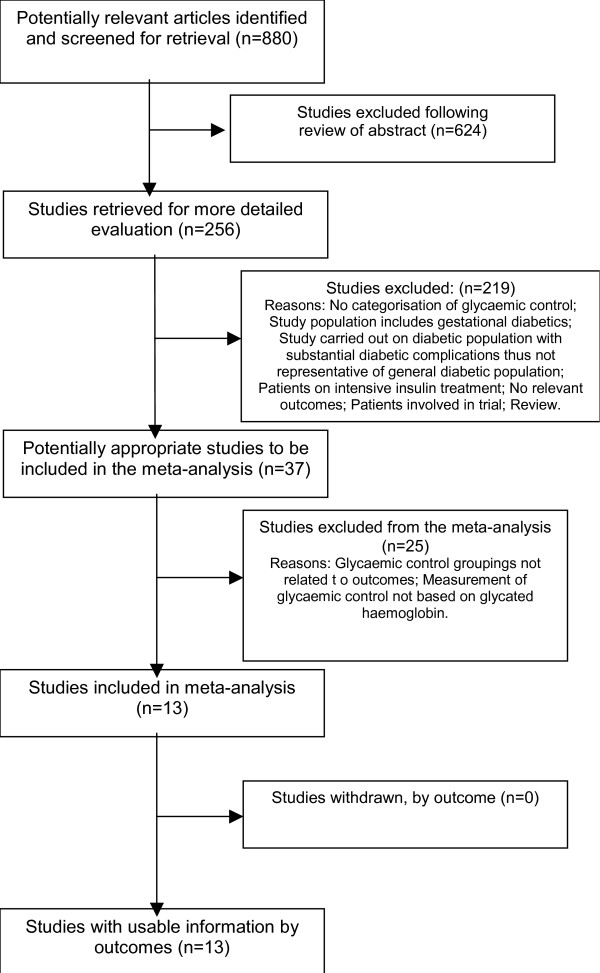
Flow of studies in the review.

Our study identified 880 published studies from our search strategy. We retrieved the text of 256 and reviewed them to assess whether they provided information on HbA_1c _and adverse pregnancy outcome in pregnant women with type 1 and type 2 diabetes. After we applied all exclusion and inclusion criteria, thirteen studies which compared poor vs. optimal glycaemic control in relation to maternal, fetal and neonatal outcomes, were included in this review, (Figure 2 contains the flow diagram of studies). Of these, eight were cohorts, two case-controls, one cross-sectional and two historical reviews.

### Qualitative summary

Seven studies specifically looked at only women with Type 1 diabetes and the remaining six studies included both Type 1 and Type 2. Table 1 summarises the characteristics of all studies included in the analysis. Study populations were from United States, United Kingdom, Finland, France, Netherlands, Sweden and Poland. Sample sizes ranged from 83 to 2459 participants in the largest study, with a total of 5480 women. Most studies involved patients who were receiving pregnancy care at the study outpatient clinics. All studies described basic inclusion and exclusion criteria for study participants.

**Table 1:** Design characteristics of observational studies of glycated haemoglobin and pregnancy outcomes.

***Author***	***Country***	***Study Design***	***Time Period***	***Sample size (n)***	***Glycaemic Control Groupings (n) (HbA_1c _unless stated)***	***Timing of Glycaemic Control Measurement***	***Outcomes (n)***
Vaarasmaki *et al*, 2000 [26]	Finland	Cohort	1986–1995	84	Optimal < 8.0% (n = 48) Poor > 8.0% (n = 36)	First antenatal visit 20 and 28^th ^week Before delivery	Malformations (n = 4) Caesarean section (n = 34) Stillbirth (n = 1) NICU (n = 39) Neonatal hypoglycaemia(n = 11) RDS (n = 5)
Greene *et al*, 1989 [23]	US	Cohort	Dec.5^th ^1983–Dec.31^st ^1987	303	Optimal ≤ 9.3% (n = 113) Poor ≥ 9.4% (n = 190) (Data based on HbA_1_)	1^st ^trimester	Malformations (n = 20) Spontaneous abortion (n = 52)
Evers *et al*, 2004 [16]	Netherlands	Cohort	April 1^st ^1999 – April 1^st ^2000	261	Optimal ≤ 7.0% (n = 212) Poor ≥ 7.0% (n = 71)	1^st ^trimester	Malformations (n = 29)
Key *et al*, 1987 [19].	US	Cohort	Jan.1^st ^1979 – Dec.31^st ^1984	83	Optimal <7.5% (n = 8) Poor ≥ 7.5% (n = 75)	1^st ^trimester	Malformations (n = 9) Spontaneous abortion (n = 22)
Temple *et al*, 2002 [7]	UK	Cohort	Jan. 1991–Dec. 2000	158	Optimal < 7.5% (n = 110) Poor ≥ 7.5% (n = 48)	1^st ^trimester booking	Malformations (n = 5) Stillbirth (n = 2) Spontaneous abortion (n = 11) Neonatal death (n = 2)
Kitzmiller *et al*, 1991 [22]	USA	Cohort	1982–1988	194	Optimal ≤ 7.6% (n = 53) Poor > 7.6% (n = 141)	1^st ^trimester booking visit	Malformations (n = 13)
Ylinen *et al*, 1984 [21]	Finland	Cohort	April 1978–Dec. 1982	142	Optimal ≤ 7.9% (n = 63) Poor >7.9% (n = 79)	Before 16 weeks gestation	Malformations (n = 17)
CEMACH, 2005 [25]	England, Wales & Northern Ireland	Descriptive Cohort	March 1^st ^2002–Feb 28^th ^2003	2459	Optimal < 7% (n = 962) Poor ≥ 7% (n = 1497)	1^st ^trimester	Malformations (n = 101) Stillbirths or neonatal deaths (n = 67)
Hiilesmaa *et al*, 2000 [17]	Finland	Case-Control	1988 – 1997	587	Optimal ≤ 6.8% (n = 195) Poor > 6.8% (n = 392)	Early pregnancy	Pre-eclampsia (n = 77) PIH (n = 65)
Hanson *et al*, 1990 [24]	Sweden	Case-Control	1982–1985	532	Optimal < 10.1% (n = 490) Poor ≥ 10.1% (n = 42)	1^st ^trimester	Spontaneous abortion (n = 41) Malformation (n = 21)
Diabetes and Pregnancy Group, France 2003 [15]	France	Cross-sectional	Jan. 2000 – Dec. 2001	435	Optimal < 8.0% (n = 315) Poor > 8.0% (n = 120)	1^st ^trimester	Malformations (n = 18) Perinatal mortality (n = 19) Preterm delivery (n = 147)
Miller *et al*, 1981 [18]	US	Case-series	April 1977 – April 1980	116	Optimal ≤ 8.5% (n = 58) Poor ≥ 8.6% (n = 58)	Initial value	Malformations (n = 15)
Wender-Ozegowska *et al*, 2005 [20]	Poland	Case-Series	1^st ^Jan. 1994–31^st ^Jan. 1999	126	Optimal ≤ 5.6% (n = 43) Poor > 5.6% (n = 83)	1^st ^trimester	Malformations (n = 14)

The method of measurement of HbA_1c _varied across all the studies. Five studies used high-performance liquid chromatography [7,15-18]. Other methods used included spectrophotometric absorption, [19] column chromatography, [20] cation exchange method, [21] thiobarbituric acid colorimetric assay, [22] electrophoresis, [23] and isoelectric focusing [24]. Two of the studies did not give details on their method of measuring HbA_1c _[25,26].

All studies used different cut-off points for grouping HbA_1c _into poor and optimal groups, varying from 5.6% to 10.1%. The timing of the glycaemic control measurement varied across the studies, the majority of studies (twelve) measured HbA_1c _during the first trimester. One study based glycaemic control on measurements taken at the first antenatal visit, 20^th ^and 28^th ^week of gestation, and just before delivery [26].

The data extraction method varied across the studies and very few studies [15,17]. adjusted for potential confounding factors in their analysis, (Table 2). Of these, neither stated what specific factors they adjusted for in the analysis.

**Table 2:** Quality assessment of included studies.

**Authors Reference**	**26**	**23**	**16**	**19**	**7**	**22**	**21**	**25**	**17**	**24**	**15**	**18**	**20**
Is the hypothesis clearly defined	Y	Y	Y	Y	Y	Y	Y	Y	Y	Y	Y	Y	Y
Inclusion criteria defined	Y	Y	Y	Y	N	Y	Y	Y	Y	Y	Y	Y	Y
Method of sample selection stated	Y	Y	Y	Y	Y	Y	Y	Y	Y	Y	Y	Y	Y
Data extraction method stated	Y	Y	Y	Y	N	Y	Y	Y	Y	Y	Y	Y	Y
Adequate description of diagnostic criteria	Y	Y	Y	Y	N	Y	Y	Y	Y	Y	Y	N	Y
Clinical and demographic characteristics fully defined	Y	Y	Y	Y	Y	Y	Y	Y	Y	Y	Y	Y	Y
Complete and representative sample of patients	Y	Y	Y	Y	Y	Y	Y	Y	Y	Y	Y	Y	Y
Appropriate follow-up of patients	Y	Y	Y	Y	Y	Y	Y	Y	Y	Y	Y	Y	Y
Losses to follow up	N	Y	Y	Y	N	Y	NA	N	NA	Y	NA	NA	NA
Unbiased outcome	Y	Y	Y	Y	Y	Y	Y	Y	Y	Y	Y	Y	Y
Fully defined outcome	Y	Y	Y	N	N	Y	Y	Y	Y	Y	Y	Y	N
Appropriate outcome	Y	Y	Y	Y	Y	Y	Y	Y	Y	Y	Y	Y	Y
Outcome known for all or a high proportion of patients	Y	Y	Y	Y	Y	Y	Y	Y	Y	Y	Y	Y	Y
Fully defined prognostic variable	Y	Y	Y	Y	Y	Y	Y	Y	Y	Y	Y	Y	Y
Prognostic variable available for all or a high proportion of patients	N	Y	Y	Y	Y	Y	Y	N	Y	Y	Y	Y	Y
Continuous predictor variable analysed appropriately	Y	Y	Y	Y	Y	Y	Y	Y	Y	Y	Y	Y	Y
Statistical adjustment for all important prognostic variables	N	N	N	N	N	N	N	NA	Y	N	Y	N	N

### Quantitative summary

The most common pregnancy outcome to be investigated was the outcome of congenital malformation, in which twelve out of the thirteen studies reported, (Table 3). Spontaneous abortions and perinatal mortality were reported in four of the studies, while all other maternal, fetal and neonatal outcomes in Table 3 were reported in only one of the studies.

**Table 3:** Pregnancy outcomes and number of studies included in review reporting outcome.

**Outcome**	**No. of studies reporting outcomes**
Congenital malformations	12
Spontaneous abortions	4
Perinatal mortality	4
Neonatal deaths	1
Preterm delivery	1
Neonatal hypoglycaemia	1
Neonatal Intensive Care Unit Admission	1
Respiratory Distress Syndrome	1
Caesarean Section	1
Pre-eclampsia	1
Pregnancy-induced hypertension	1

**Figure 3:**
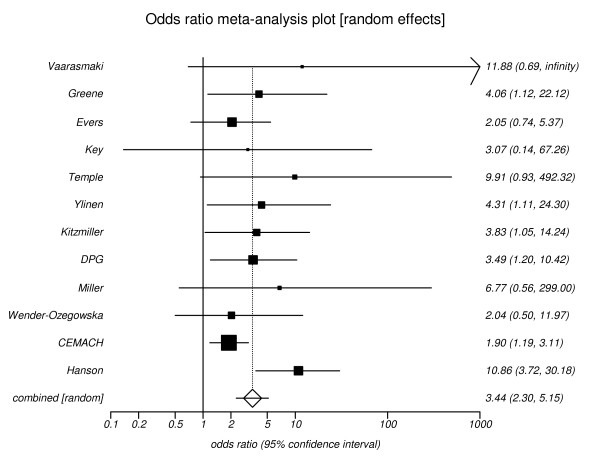
**Risk of congenital malformation for poor versus optimal glycaemic control**. Pooled odds ratio = 3.44 (95% CI = 3.00 to 5.15). Chi^2 ^(test odds ratio differs from 1) = 36.2 (df = 1) P < 0.001.

**Figure 4:**
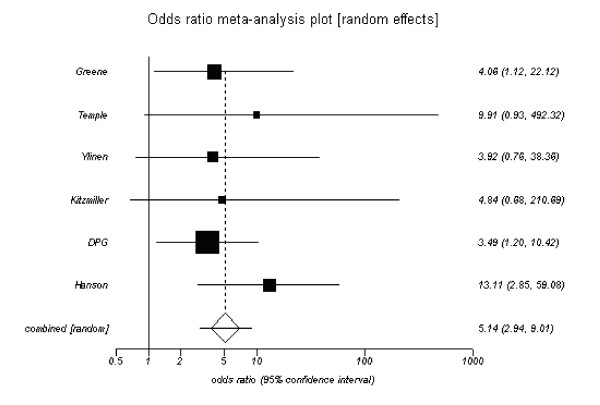
**Risk of major congenital malformation for poor versus optimal glycaemic control**. Pooled odds ratio = 5.14 (95% CI = 3.00 to 9.01). Chi^2 ^(test odds ratio differs from 1) = 32.8 (df = 1) P < 0.001.

The pooled estimate for patients with poor control and the outcome of congenital malformations was 3.44 (95% CI, 2.30 to 5.15), (Figure 3). Six studies only reported major congenital malformations and the pooled estimate was 5.14 (95% CI, 2.94 to 9.01), (Figure 4). It was possible to calculate a relative risk for each 1-percent point increase in HbA_1c _for four out of the twelve studies which investigated the outcome of congenital malformations, these are presented in Table 4. The relative risk estimates varied from 1.63 to 2.34 per 1-percent point increase in HbA_1c_. These could be translated to a relative risk reduction per 1-percent point decrease in HbA_1c _and varied from 0.39 to 0.59.

**Table 4:** Relative risk estimates per 1-percentage point increase in glycated haemoglobin and the outcome of congenital malformation.

**Author**	**Mean**	**SD**	**25^th ^percentile**	**75^th ^percentile**	**Difference between 25^th ^and 75^th ^percentile**	**Inverse Relative Risk**	**Relative Risk per 1% point increase**	**Relative Risk per 1% point decrease**
Miller [18]	8.54	1.54	7.50	9.58	2.08	1.87	2.34	0.59
Greene [23]	10.10	1.99	8.76	11.44	2.68	1.31	1.63	0.39
Evers [16]	6.5	0.70	6.03	6.97	0.94	0.65	1.99	0.50
Key [19]	10.99	1.10	10.25	11.73	1.48	1.0	1.95	0.49

**Figure 5:**
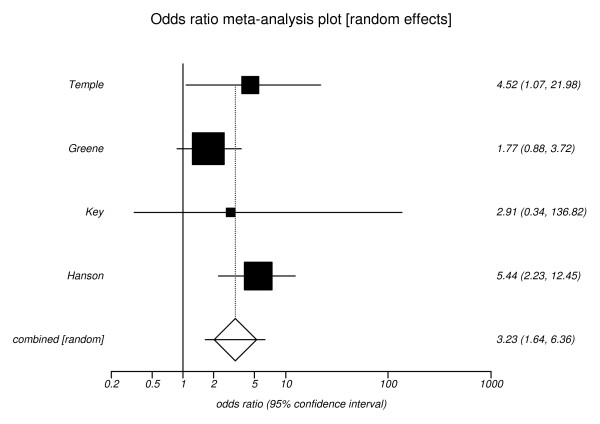
**Risk of miscarriage for poor versus optimal glycaemic control**. Pooled odds ratio = 3.23 (95% CI = 1.64 to 6.36). Chi^2 ^(test odds ratio differs from 1) = 11.48 (df = 1) P = 0.001.

**Figure 6:**
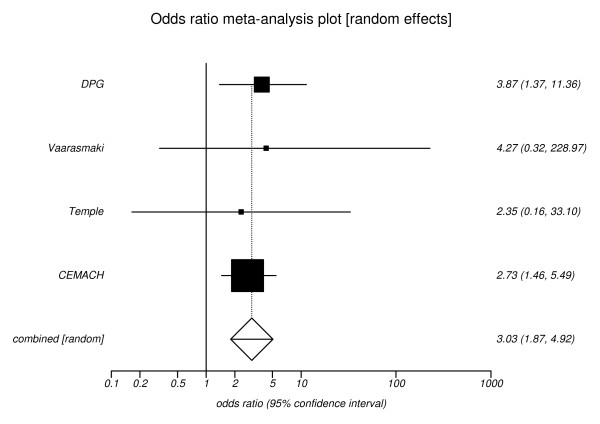
**Risk of perinatal mortality for poor versus optimal glycaemic control**. Pooled odds ratio = 3.03 (95% CI = 1.87 to 4.92). Chi^2 ^(test odds ratio differs from 1) = 20.13 (df = 1) P < 0.0001.

The pooled estimate for the outcome of miscarriage was 3.23 (95% CI, 1.64 to 6.36), (Figure 5) and for the outcome of perinatal mortality an odds ratio of 3.03 (95% CI, 1.87 to 4.92), (Figure 6).

Sensitivity analyses indicated that three of the studies seemed to contribute more greatly to the analysis [15,16,25]. Sensitivity analyses, excluding the study with the largest number of participants [25], produced similar results.

### Publication bias

**Figure 7:**
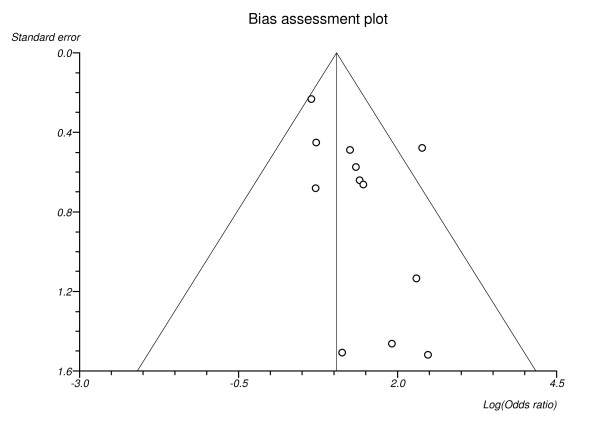
**Bias assessment plot for the outcome of congenital malformations**. Begg-Mazumdar: Kendall's tau = 0.33 P = 0.15. Egger: bias = 1.27 (95% CI = -0.01 to 2.56) P = 0.05. Horbold-Egger: bias = 2.19 (95% CI = 0.53 to 3.84) P = 0.03.

A bias assessment plot for the outcome of congenital malformations is shown in Figure 7. The Egger test was not significant (*P *> 0.05) for the congenital malformation subgroup analysis. For the other outcomes however, the small number of studies limits our ability to draw conclusions regarding publication bias and heterogeneity of studies.

## Discussion

In our analysis of thirteen published studies, adverse pregnancy outcomes were associated with higher levels of HbA_1c _in women with type 1 and type 2 diabetes mellitus. These associations were present across different geographic populations and different time periods. A decrease in HbA_1c _was associated with a clinically important reduction in the risk of fetal congenital malformations.

The outcome of congenital malformations was the most commonly reported outcome across the studies. Reasons for this may be because many clinical and epidemiological studies indicate that fetal malformations in pregnancy complicated by diabetes are due to metabolic disturbances affecting the process of organogenesis, which takes place at the early stage of pregnancy. The most common malformations in infants of mothers with diabetes are defects of the cardiovascular system. Studies suggest that these tissues are the most susceptible ones to the destructive action of oxygen-free radicals [20]. Our analysis shows that poor glycaemic control is associated with a greater than three-fold risk for the outcome of congenital malformations compared to optimal glycaemic control. The rates of miscarriage and perinatal mortality were higher in the poor glycaemic control groups compared with the optimal control groups. Perinatal mortality rates have markedly decreased over the last 25 years, however there still appears to be a higher rate of perinatal mortality with poorer levels of glycaemic control.

There are limited randomised trial data on the impact of different levels of glycaemic control on outcome in diabetic pregnancies [27]. The randomised, prospective Diabetes Control and Complications Trial (DCCT) has shown that timely institution of intensive therapy for blood glucose control is associated with rates of spontaneous abortion and congenital malformations that are similar to those in the non-diabetic populations [28]. A Cochrane systematic review of randomised trials comparing very tight with tight control of diabetes in pregnancy focused on two trials involving a total of 182 women. The conclusion was that there was no clear evidence of benefit from very tight versus tight glycaemic control for pregnant women with diabetes [27]. Observational studies show much less favourable outcomes in unselected populations. In many studies, adverse outcomes remain more common among the infants of mothers with type 1 diabetes than in the general population [8,29]. The targets of the St. Vincent's Declaration of 1989 appear not to have been met, thus far. Reasons for persistently poor outcomes in these populations may include unplanned pregnancies, pregnancies in women who have not received pre-conceptual care, or pregnancies in women who fail to achieve optimal control despite adequate pre-conceptual care.

The factors that influence women to seek preconception care and counselling and then to actually achieve optimal glycaemic control prior to conception have become important to clinicians. Factors that seem to promote preconception care include higher educational levels, higher incomes, regular employment, and receiving encouragement from their health care providers to avoid unplanned pregnancies [30]. Past pregnancy experience may also play a role through influencing behaviour concerning diabetic control and health habits [31].

A systematic review of 14 cohort studies has shown that pre-conception care aiming to achieve tight glycaemic control is associated with a reduction in the rate of major congenital abnormalities – 2.1% in the preconception care recipients versus 6.5% in non recipients, relative risk 0.36, 95% CI 0.22 to 0.59 [32].

Patients who frequently monitor and adjust their diabetes regimen are more likely to maintain strict control of their blood glucose levels throughout pregnancy [33]. Our findings support this with a marked increase in congenital malformation in association with poor glycaemic control. A decremental approach to HbA_1c _may appeal to women who are overwhelmed at the prospect of achieving a dramatic change in control from poor to optimal. Advising women that there is a potential health gain with each 1% reduction may be a useful motivator in gradual reduction to an optimal level or may provide some reassurance for women who manage a large improvement but do not quite achieve optimal levels.

This review has several limitations. It is unclear to what extent methodologic limitations, such as residual confounding and selection bias, might exist in these studies. The pooled odds ratios have been used to quantify the risks, however the small number of studies meant that statistical analyses for heterogeneity and publication bias were limited. Although the funnel plot for the outcome of congenital malformation shows an indication of asymmetry, with just twelve studies the power to detect asymmetry in a funnel plot is low. Thus, we cannot make any conclusions about publication bias and we cannot exclude the possibility that the observed association is a result of publication bias. We believe our process of literature identification was comprehensive and captured all of the published studies on the relation between HbA_1c _and outcomes in pregnant women with diabetes.

The studies use different definitions of poor and optimal control, ranging from 5.6% to 10.1%. Reasons for this include the use of different methods of measurements for HbA_1c _and varying reference ranges for the non-diabetic population. Nonetheless, the cut-offs used were appropriate to the method used to measure HbA_1c _and relevant to the reference range in use for the individual study populations. For this reason it would have been inappropriate to do a subgroup analysis using different cut-off levels as categories.

Definitions for several of the outcomes varied across the studies, for example, the outcome of congenital malformations included both major and minor malformations in some studies [16,26] while in others only included major malformations [7,18].

Few studies adjusted for confounding factors in their analysis and there is no certainty that the observed association was caused exclusively by an elevated HbA_1c _level rather than to some degree by related confounders. In the majority of the studies we do not know how advanced the patients' diabetes was. Diabetic nephropathy and retinopathy are the most frequent complications in patients of childbearing age with diabetes and will have an important impact on pregnancy outcome [34]. One possible causal factor for adverse outcome could be women with established diabetes complications, such as microvascular disease. A single unsatisfactory HbA_1c _value cannot be used as an absolute predictor of fetal outcome, but it indicates a subgroup of pregnancies with substantial fetal risk [26].

Major advantages of pooling data from observational studies to investigate this important clinical issue are better generalisability because the analyses combine data from heterogeneous populations, and increased sample size.

## Conclusion

Our systematic review highlights important weaknesses in the literature. Studies to date are based on very small numbers and this systematic review allows more robust estimate of risk. Many important clinical outcomes were not examined in the thirteen studies included in the review. More than a decade after the initial evidence proposing that pregnancy outcome was improved by better glycaemic control, the question of how strict that control should be remains unanswered. There remains an urgent need to address the maternal and perinatal benefits of varying degrees of control of blood sugar for pregnant women with diabetes. Outcome measures should be standardised and include important factors associated with poor perinatal and maternal outcomes, such as pre-eclampsia, macrosomia, caesarean section, shoulder dystocia, perinatal loss, neonatal respiratory and metabolic complications [27]. Future studies also need to investigate the issue of pre-conceptual glycaemic control and post pregnancy outcomes for the mother. We are currently undertaking a study exploring the related issues of pre-conceptual glycaemic control, antenatal care, and mode of delivery in terms of pregnancy-related, maternal and neonatal outcomes both in the short- and long-term.

We conclude that adverse pregnancy outcomes in women with type 1 and type 2 diabetes mellitus were associated with higher levels of HbA_1c_. This review summarises the currently available evidence and should be useful to clinicians who are counselling women with type 1 and type 2 diabetes in the reproductive years.

## Competing interests

The author(s) declare that they have no competing interests.

## Authors' contributions

MEI, TPF and DJM conceived the review, MEI reviewed and analysed the data and wrote the review. All authors interpreted the data, contributed to writing the manuscript, and gave critical comments. All authors have given approval of the final version to be published.

## Pre-publication history

The pre-publication history for this paper can be accessed here:



## Supplementary Material

Additional file 1 QUORUM statement. Click here for file
