# Supplementary material for: Poor glycated haemoglobin control and adverse pregnancy outcomes in type 1 and type 2 diabetes mellitus: Systematic review of observational studies
Source: BMC Pregnancy Childbirth. 2006 Oct 30;6:30. doi: 10.1186/1471-2393-6-30 (PMC1635059; doi:10.1186/1471-2393-6-30)
Supplement: Additional file 1 — QUORUM statement. [file 1471-2393-6-30-S1.pdf]

Improving the quality of reports of meta-analyses of randomised controlled trials: the QUOROM statement checklist

Heading	Subheading	Descriptor	Reported? (Y/N)	Page number
Title		Identify the report as a meta-analysis [or systematic review] of RCTs[26]	Y	1
Abstract		Use a structured format[27]	Y	2
		**Describe**		
	Objectives	The clinical question explicitly	Y	2
	Data sources	The databases (ie, list) and other information sources	Y	2
	Review methods	The selection criteria (ie, population, intervention, outcome, and study design); methods for validity assessment, data abstraction, and study characteristics, and quantitative data synthesis in sufficient detail to permit replication	Y	2
	Results	Characteristics of the RCTs included and excluded; qualitative and quantitative findings (ie, point estimates and confidence intervals); and subgroup analyses	Y	2
	Conclusion	The main results	Y	3
		**Describe**		
Introduction		The explicit clinical problem, biological rationale for the intervention, and rationale for review	Y	4
Methods	Searching	The information sources, in detail[28] (eg, databases, registers, personal files, expert informants, agencies, hand-searching), and any restrictions (years considered, publication status,[29] language of publication[30,31])	Y	5
	Selection	The inclusion and exclusion criteria (defining population, intervention, principal outcomes, and study design[32]	Y	5
	Validity assessment	The criteria and process used (eg, masked conditions, quality assessment, and their findings[33–36])	Y	6
	Data abstraction	The process or processes used (eg, completed independently, in duplicate)[35,36]	Y	6
	Study characteristics	The type of study design, participants' characteristics, details of intervention, outcome definitions, &c,[37] and how clinical heterogeneity was assessed	Y	6
	Quantitative data synthesis	The principal measures of effect (eg, relative risk), method of combining results (statistical testing and confidence intervals), handling of missing data; how statistical heterogeneity was assessed;[38] a rationale for any a-priori sensitivity and subgroup analyses; and any assessment of publication bias[39]	Y	6,7
Results	Trial flow	Provide a meta-analysis profile summarising trial flow (see figure)	Y	25
	Study characteristics	Present descriptive data for each trial (eg, age, sample size, intervention, dose, duration, follow-up period)	Y	8,9,20
	Quantitative data synthesis	Report agreement on the selection and validity assessment; present simple summary results (for each treatment group in each trial, for each primary outcome); present data needed to calculate effect sizes and confidence intervals in intention-to-treat analyses (eg 2×2 tables of counts, means and SDs, proportions)	Y	9,10,26-30
Discussion		Summarise key findings; discuss clinical inferences based on internal and external validity; interpret the results in light of the totality of available evidence; describe potential biases in the review process (eg, publication bias); and suggest a future research agenda	Y	11-15

Quality of reporting of meta-analyses
